# Malaria mortality in Colombia from 2009 to 2018: a descriptive study

**DOI:** 10.1590/0037-8682-0441-2020

**Published:** 2021-02-26

**Authors:** Shirley Natali Iza Rodríguez, José Alejandro Iza Rodríguez, Julio Cesar Padilla Rodríguez, Mario Javier Olivera

**Affiliations:** 1 Universidad Militar Nueva Granada, Department of Medicine, Bogotá, Colombia.; 2 Universidad Nacional de Colombia, Department of Medicine, Bogotá, Colombia.; 3 Ministerio de Salud y Protección Social, Bogotá, Colombia.; 4 Instituto Nacional de Salud, Parasitology Team, Bogotá, Colombia.

**Keywords:** Malaria, Death certificates, Mortality rate, Epidemiology, Colombia

## Abstract

**INTRODUCTION:**

Colombia has an endemo-epidemic for malaria, with a downward trend in mortality over the last few decades. This study describes the malaria mortality rates from 2009-2018.

**METHODS:**

We obtained data from the Colombian Mortality Information System and calculated the case fatality and crude and age-adjusted mortality rates.

**RESULTS:**

During the study, 148 malaria-related deaths were registered. The average annual mortality rate was 0.032 deaths/100,000. Two peaks were observed in 2010 and 2016. Choco contributed to the highest number of deaths (27.7%).

**CONCLUSIONS:**

The unstable downward trend of malaria mortality rates calls for greater emphasis on surveillance and interventions.

The risk of contracting malaria is a significant health issue in approximately 100 tropical, subtropical, and temperate countries, with an estimated rate of 228 million cases and approximately 405,000 deaths worldwide in 2018 according to the World Health Organization (WHO). The incidence rate declined from 71 to 57 per 1000 inhabitants at risk, and the total number of cases was reduced by 9.6% between 2010 and 2018 globally. During the same period, the global mortality, which has declined since 2016, showed a reduction of 30%^1^. 

The current distribution of human-pathogenic *Plasmodium* species shows a predominance of *P. vivax* in the Americas and *P. falciparum* in Africa and the Mediterranean region, whereas both the species are prevalent in Asia. Other species are widespread with a generally low prevalence^1^ ^,^ ^2^. Colombia, situated in South America, shows variable endemic-epidemic patterns among its various regions with high prevalences of *P. vivax*, except some regions (Chocó, Nariño, Cauca, and Vaupes) where a predominance of *P. falciparum* can be observed. The endemicity in Colombia is low and unstable compared to that in African countries, with a downward trend in reported cases from 171,960 in 2000 to 63,143 in 2018, which represents a reduction of 63%^3^. This trend was maintained with some fluctuations, except two peaks: in 2010 with 117,638 cases, increasing by 32% compared to the previous year^4^, and in 2016 with 83,356 cases^5^.

Most of the urban areas in Colombia lie along the Andean Mountains where the conditions are not favorable for malarial transmission, which is in contrast with the majority of the rural territory where physical (climate, altitude), and also social, cultural, and even economic factors (mining and deforestation) favor the transmission and/or disturbance of control program effectiveness^6^ ^,^ ^7^. Of special mention is the Pacific region (Cauca, Valle del Cauca, Choco, and Nariño), which is a highly endemic region for malaria and one of the least economically developed regions in Colombia, with Afro-descendants comprising most of the population. Hence, a huge disease burden lies on this ethnicity. 

According to the Ministry of Health and Social Protection, approximately 10 million people in Colombia were at risk of infection in 2019, representing 22% of the national population^8^. Most malaria cases were confined to 70 municipalities, 53 of which have approximately 2 million inhabitants, which are classified as high risk with annual parasite indices (API) higher than 10 cases per 1000 residents^3^ ^,^ ^6^. 

Furthermore, the mortality rate maintained a decreasing trend with fluctuations and plateaus. Between 1979 and 2008, 6,965 deaths due to malaria were reported in Colombia. The age-specific mortality rate decreased in all groups, and it was more pronounced in the extreme ages^9^. Among pregnant women, low prevalences of infection and mortality have been observed in Latin America, where most cases present as uncomplicated malaria with low parasitemia. The presence of severe organ dysfunction showed no dependence on the parasite species, bringing into question the general consideration of *P. vivax* as benign^10^.

According to a 2006 study by Valero and the WHO report in 2017, Colombia has had a constant annual blood examination rate below 6% since 1960 and had registered one of the lowest rates during the 2016 peak, it being well below the 10% recommended minimum. This suggested that the diagnostic monitoring activity should be improved and data regarding the malaria burden should be treated with caution^11^ ^,^ ^12^. Although the data may contain a degree of underestimation, similar downturns registered in Brazil and Guyana suggest that the data are reliable and that Colombian programs on vector-control, coverage, access to health care, and antimalarial treatments have been effective^8^ ^,^ ^13^. 

The present study describes the trend of malaria mortality in Colombia from 2009 to 2018. A descriptive study was conducted using national mortality records from the National Administrative Department of Statistics (DANE) of the period 2009-2018. The records included all deaths that occurred and were registered in the country, with data regarding sex and 5-year age group. These records were in the public domain and were freely available on the website of the DANE. The variable of interest was mortality from malaria, defined by the basic cause of death using the tenth revision of the International Classification of Diseases with the following codes: P373, P374, B500, B508-B510, B518-B520, B528-B531, B538, and B54x. Additionally, demographic information was obtained from population projections and series from the DANE webpage for the same period^14^. Publicly available information on malaria cases was obtained from the morbidity records of the National Public Health Surveillance System of the National Institute of Health for the years 2009-2018.

Descriptive statistics for the study population included the calculation of absolute numbers and proportions (with 95% confidence intervals [95% CI]). A direct standardization method was applied to eliminate the effect of age on mortality by using the Colombian population from the 2005 census as the standard. To calculate the expected number of deaths, the annual death rate for each 5-year age group of the general population was multiplied by the number of malaria cases in the same age group. The standardized mortality ratio (SMR) was calculated as the ratio between the deaths observed in the study group and the expected mortality in the general population and was expressed per 100,000 inhabitants. The crude mortality rate (CMR) and malaria case fatality rate (mCFR) were also calculated. The mCFR was evaluated as the proportion of the total number of malaria deaths divided by the total number of severe malaria cases (confirmed). All the data were stored in a standard format in MS Excel (Microsoft, Redmond, USA) and analyzed using Stata (release 15, Stata Corporation, College Station, TX, USA). 

From 2009 to 2018, a total of 6,854 cases of severe malaria and 148 malaria-related deaths were registered in Colombia, of which 51.4% (76) were women, and of these, 34% were within childbearing age. The male-to-female sex ratio was 1: 1.05. The average SMR in the period was 0.032 deaths/100,000 inhabitants and the overall mCFR was 2 deaths for 100 severe malaria cases. There was a decreasing trend in mCFR between 2009 and 2018. The mCFR went from 6 (95% CI 4.9-7.1%) to 1 (95% CI 0.8-1.2%) deaths for 100 severe malaria cases in 2009 and 2018, respectively. The average annual age-adjusted mortality rates were similar in males and females (0.034 versus 0.030 deaths/100,000). The highest age-specific mortality rates were found in the young age groups, especially within 15-44 years (0.012 deaths/100,000) (Table 1). In this study period, *P. falciparum* infections predominated with 57.4% cases (85 cases) and an SMR of 0.017, whereas *P. vivax* had an SMR of 0.013. Except in the years 2010-2012, despite *P. vivax* being endemic to Colombia, *P. falciparum* caused the greatest number of infections due to its higher mortality (mCFR of 2.5 and 1.8 deaths for 100 severe malaria cases in *P. falciparum* and *P. vivax malaria,* respectively), particularly in Choco. In 2010 and 2016, the number of deaths was well above the mean SMR of the current decade (0.053), corresponding with the epidemiological alarm from the WHO concerning a burst of malaria cases. The 2010 peak represented a 27% increase in mortality compared to the 2009 rate. This increased mortality was more evident in Choco, which traditionally has the highest rate of transmission in Colombia. The usual preponderance of *P. falciparum* in Choco presented a gradual reversal to higher *P. vivax* mortality rates in the 2010-2012 period. During the 2016 peak, a 62% increase from the previous year was registered with the expected predominance of *P. falciparum* (Figure 1). The general SMRs in the 2010 and 2016 peaks were 0.052 and 0.053, respectively. 

**TABLE 1:** Epidemiological characteristics including sex-, *Plasmodium* species- and age-adjusted malaria-related mortality rates (per 100,000 inhabitants) in Colombia, 2009-2018.

Variable	Total deaths (%)	Mortality rates (per 100,000 inhabitants) (95% CI)	***P* value**
**All malaria-related deaths**	148 (100)	0.0322 (0.022 - 0.041)	
**Sex**			
Male	72 (48.6)	0.0300 (0.028 - 0.031)	Ref
Female	76 (51.4)	0.0349 (0.033 - 0.036)	0.3992
**Age group (years)**			
<5	15 (10.1)	0.0031 (0.000 - 0.005)	Ref
5-14	22 (14.9)	0.0046 (0.001 - 0.007)	0.2076
15-44	59 (39.9)	0.0125 (0.008 - 0.016)	0.0030
45-64	26 (17.6)	0.0055 (0.002 - 0.008)	0.2048
≥65	26 (17.6)	0.0054 (0.003 - 0.007)	0.0166
***Plasmodium* species**			
*P. vivax*	63 (42.6)	0.0133 (0.009 - 0.017)	Ref
*P. falciparum*	85 (57.4)	0.0174 (0.009 - 0.025)	0.2812

*Ref: reference.

**FIGURE 1:**
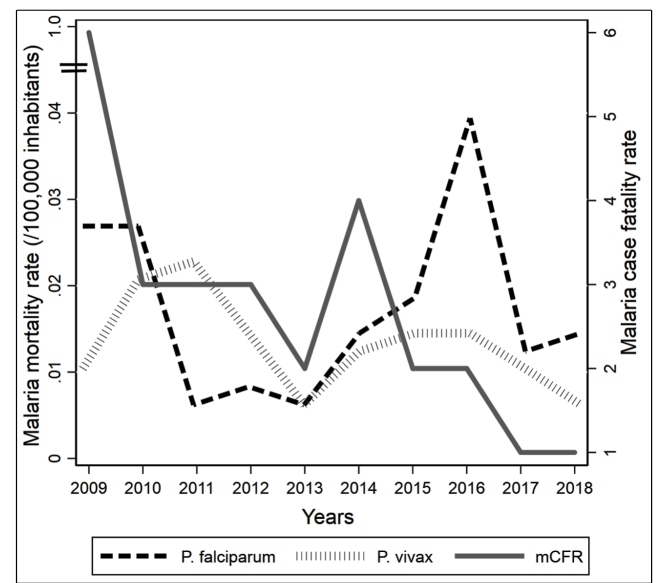
Standardized mortality and case fatality rates due to malaria by *Plasmodium* species, 2009-2018. **mCFR**: Malaria case fatality rate.

Throughout the study period, Choco and Antioquia together registered 43% of the overall mortality. Other regions that contributed significantly to mortality rate were Valle del Cauca, Cordoba, and Nariño (Figure 2). Further, Choco and Antioquia represented 37% of the out-of-hospital deaths recorded. Higher mortality numbers were seen in age groups <5 years and young adults. Job occupation information was available for only 45% of the registrants. Of those, 38% were housekeepers; 20%, students; 9%, farmers; 6%, military employees; 1.49%, miners; and, 23%, others.

**FIGURE 2:**
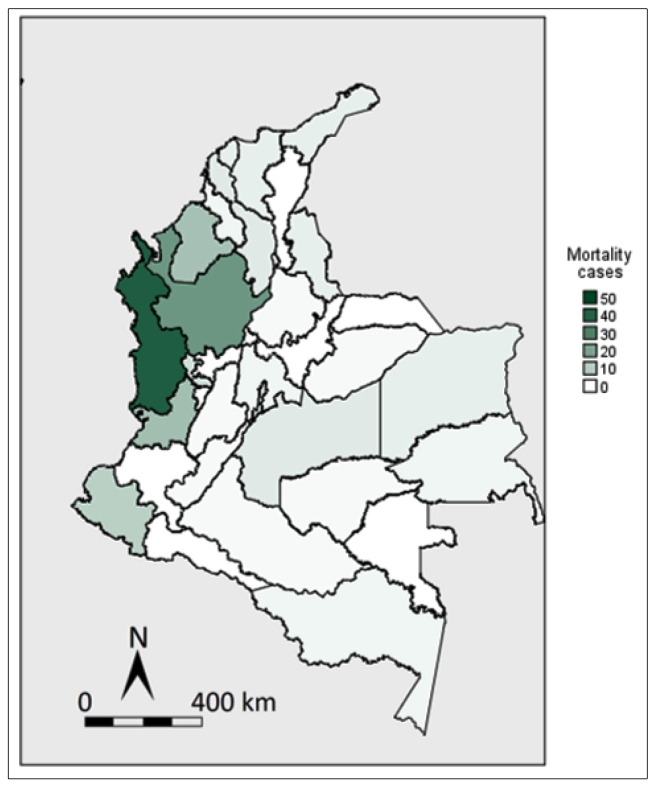
Malaria mortality by departments in Colombia, 2009-2018

The highest mortality rate was registered in the young-adult population, which is economically active. Also noteworthy is the high mortality rate in children under 5 years of age. The high mortality rate in women of childbearing age (34%) also represents a major issue. Based on these data, malarial transmission around or inside schools and houses may be suspected. Approximately 27% of the mortality cases corresponded to Afro-descendants and 23% to the indigenous people; the information on ethnicity was unavailable for the remaining cases. Of the 148 registrants, only 45.9% had complete information; the remaining 54% lacked some data (ethnicity, occupation, parasite species, and others). This finding can be explained by the inadequacy of the training given to the people in charge of filling the databases, omission, and the reluctance of the affected relatives to provide information due to having lived in areas of armed conflict or illegal economy.

Based on the results of this study, the malaria-related mortality rate in Colombia between 2009 and 2018 was variable, with no sustained decreases and the presence of epidemic bursts. As expected, the most endemic departments presented the greatest number of cases throughout the studied decade. Despite incomplete reporting, the rates of Afro-descendants and indigenous people together accounted for approximately half of the mortality, with higher rates in 2010 and 2016. Out-of-hospital deaths were notable in the departments with the largest burdens, i.e., Choco, Nariño, and Antioquia.

In most years, mortality due to *P. falciparum* was higher than that due to other species in Choco, except for the outstanding reversal during 2010-2012 when *P. vivax* accounted for more deaths.

Parasitological identification was present in all cases except for one, which demonstrates important improvements in diagnostic and notification procedures as compared to prior studies where the absence of this information was much more common. 

Certain occupational activities have shown correlations with malaria rates; however, most of the cases in this study lacked information of this important patient variable. It can be inferred from the available data that people undertaking economic activities framed within informal/independent conditions that generally lacked employment relationships contributed the most to the statistics. However, underreporting of occupations represents gaps in specific public interventions.

The variable number of malaria cases between 2009 and 2018 demonstrates the need to recognize factors that can affect the accuracy of the numerator and/or denominator. It is difficult to establish the true magnitude of mortality due to limitations that include underreporting due to a disregard for laws regulating public health surveillance, a lack of physical/technological or human resources, errors during the completion of notification forms, misdiagnosis, and lack of physician consultations^15^. These limitations are also noted in this study. The results of this study contrast with the reported downward trend in malaria cases in Colombia. 

Growing optimism for malaria control in Colombia is based on statistical data that recorded a downward trend over several years, but recent data represent the need for more mitigation efforts. Surveillance should be treated as a core intervention strategy and is as important as efforts and programs for malaria elimination.
